# 

**Published:** 1908-09-19

**Authors:** 


THE MODERN MEDICAL JOURNAL.

Telephone Number: 2734 Gerrard.

NEW SERIES. No. 80, Vol. III. No. 1152. Vol. XLIV. | SATURDAY, SEPT. 19TH, 1908. | PRICE THREEPENCE

